# Four-Component Recombinant Protein–Based Vaccine Effectiveness Against Serogroup B Meningococcal Disease in Italy

**DOI:** 10.1001/jamanetworkopen.2023.29678

**Published:** 2023-08-18

**Authors:** Lorenzo Lodi, Federica Barbati, Daniela Amicizia, Vincenzo Baldo, Anna Maria Barbui, Alessandro Bondi, Claudio Costantino, Liviana Da Dalt, Lorenza Ferrara, Francesca Fortunato, Valentina Guarnieri, Giancarlo Icardi, Giuseppe Indolfi, Domenico Martinelli, Marco Martini, Maria Moriondo, Francesco Nieddu, Diego G. Peroni, Rosa Prato, Silvia Ricci, Francesca Russo, Francesca Tirelli, Francesco Vitale, Shamez N. Ladhani, Chiara Azzari

**Affiliations:** 1 Immunology Unit, Meyer Children’s Hospital IRCCS, Florence, Italy; 2 Department of Health Sciences, University of Florence, Florence, Italy; 3 Department of Health Sciences, University of Genoa, Genoa, Italy; 4 Department of Cardiac, Thoracic, Vascular Sciences and Public Health University of Padua, Padua, Italy; 5 S.C. Microbiology and Virology Laboratory, City of Health and Science, Turin, Italy; 6 Department of Health Promotion Sciences, Maternal and Infant Care, Internal Medicine and Excellence Specialties “G. D’Alessandro,” University of Palermo, Palermo, Italy; 7 Department of Woman’s and Child’s Health, Padua University Hospital, Padua, Italy; 8 Regional Epidemiology Reference Service for the Surveillance, Prevention and Control of Infectious Diseases, Local Health Unit of Alessandria, Alessandria, Italy; 9 Hygiene Unit, Department of Medical and Surgical Sciences, Policlinico Foggia Hospital, University of Foggia, Foggia, Italy; 10 Paediatric and Liver Unit, Meyer Children’s Hospital IRCCS, Florence, Italy; 11 Department of Neurofarba, University of Florence, Florence, Italy; 12 Paediatric Unit, San Donato Hospital, Arezzo, Italy; 13 Laboratory of Immunology and Molecular Microbiology, Meyer Children’s Hospital IRCCS, Florence, Italy; 14 Pediatric Clinic, Department of Clinical and Experimental Medicine, University of Pisa, Pisa, Italy; 15 Veneto Regional Directorate of Prevention, Food Safety, Veterinary, Public Health, Venice, Italy; 16 National Infection Service, Public Health England, London, United Kingdom; 17 Paediatric Infectious Diseases Research Group, St George’s University of London, London, United Kingdom

## Abstract

**Importance:**

Population-based data on the 4-component recombinant protein–based (4CMenB) vaccine effectiveness and reduction in incidence rate ratios (IRRs) are continuously needed to assess vaccine performance in the prevention of serogroup B invasive meningococcal disease (IMD).

**Objective:**

To assess the effectiveness and reduction in IRRs associated with the 4CMenB vaccine in the pediatric population in 6 regions in Italy.

**Design, Setting, and Participants:**

This retrospective cohort screening study and case-control study included data from children aged younger than 6 years in 6 highly populated Italian regions from January 1, 2006, to January 1, 2020. Participants included children younger than 6 years diagnosed with serogroup B IMD without predisposing factors. Data were collected from regional surveillance and vaccination registries and were analyzed from September 2021 to January 2022.

**Exposures:**

Routine 4CMenB vaccination, per regional vaccination programs.

**Main Outcomes and Measures:**

The main outcome was the effectiveness of the 4CMenB vaccine in the prevention of serogroup B IMD in the population of children aged younger than 6 years in 6 Italian regions. The percentages of vaccine effectiveness (VE) were obtained through the concomitant use of a screening method and a case-control study. Secondary outcomes were the comparison of effectiveness results obtained using the 2 different computational methods, the description of serogroup B IMD incidence rates, and reduction in IRRs before and after 4CMenB introduction, as a proxy for vaccine impact.

**Results:**

The cohort screening study included a resident population of 587 561 children younger than 6 years in 3 regions with similar surveillance protocols, and the matched-case controls study assessed a resident population of 1 080 620 children younger than 6 years in 6 regions. Analyses found that 4CMenB VE in fully immunized children was 94.9% (95% CI, 83.1%-98.4%) using the screening method and 91.7% (95% CI, 24.4%-98.6%) using the case-control method. Overall reduction in IRR was 50%, reaching 70% in regions with early-start vaccination schedules. The case-control method involving 6 highly-populated Italian regions included 26 cases and 52 controls and found an estimated VE of 92.4% (95% CI, 67.6%-97.9%) in children old enough for the first vaccine dose and 95.6% (95% CI, 71.7%-99.1%) in fully immunized children. VE was more than 90% for partially immunized children. Even in regions where the first dose was administered at age 2 months, almost 20% of unvaccinated cases were among infants too young to receive the first 4CMenB dose.

**Conclusions and Relevance:**

This screening cohort study and matched case-controls study found high effectiveness of 4CMenB vaccination and greater reduction in IRR for early-start vaccination schedules in preventing invasive serogroup B meningococcal disease. The high proportion of children too young to be vaccinated among unvaccinated cases suggests that starting the vaccination even earlier may prevent more cases. Screening and case-control methods provided similar estimates of VE: either method may be used in different study settings, but concomitant use can provide more robust estimates.

## Key Points

**Question:**

What is the effectiveness associated with the 4-component recombinant protein–based (4CMenB) vaccine in preventing serogroup B meningococcal disease in pediatric populations younger than 6 years in Italy?

**Findings:**

This cohort screening study (3 regions; resident population: 587 561 children) and matched case-control study (6 regions; resident population: 1 080 620 children) of children younger than 6 years found that 4CMenB was associated with an effectiveness of more than 90% in children old enough to receive the first vaccine dose. Early-start vaccination programs were associated with reduced incidence rate ratios, and almost 20% of cases among unvaccinated children were among children too young to receive the first 4CMenB dose.

**Meaning:**

These findings suggest that new vaccination strategies should target children currently too young to be vaccinated.

## Teaser

This case-control study assess the effectiveness associated with the 4-component recombinant protein–based vaccine against meningococcal disease among children aged 6 years and younger in Italy.

## Introduction

Invasive meningococcal disease (IMD) is a life-threatening bacterial infection caused by *Neisseria meningitidis* and mainly affecting infants and young children.^1,2^ In the last 10 years, serogroup B meningococcus has been the main cause of IMD in North America and European countries, including Italy, and 1 of the most prevalent serogroups in Latin America.^3^ The highest incidence of serogroup B IMD is known to occur in the first year of life, with a peak between ages 4 and 8 months.^4,5^

The 4-component recombinant protein–based serogroup B meningococcus (4CMenB) vaccine (Bexsero, GSK), the first broadly protective serogroup B meningococcus vaccine approved for pediatric population, was licensed in the European Union in 2013,^6^ and it was implemented in the National Immunization Program in Italy in 2017.^7^ Since IMD is a rare disease, the 4CMenB vaccine was authorized on the basis of safety and immunogenicity studies that used the immunogenicity results as proxy for vaccine effectiveness (VE).^8,9^ In this situation, population-based confirmatory data regarding the effectiveness and reduction in incidence rate ratios (IRRs), as a proxy for vaccine impact, of 4CMenB vaccination are continuously needed. Currently, come evidence has been produced on this regard, including evaluation of effectiveness and reduction in IRRs of 4CMenB in 2 Italian regions (Tuscany and Veneto).^10^ All studies showed high VE and reduction in IRRs.^11,12,13,14,15,16,17,18,19^ However, due to the structural composition of the vaccine, based on nonuniversally shared variants of subcapsular meningococcal proteins, a constant monitoring of VE in different epidemiological contexts should be pursued. Moreover, effectiveness data released to date worldwide have been obtained with different methods applied simultaneously only once on a limited number of cases^16^ and claiming additional concordance validation.

This study offers the widest available perspective on the effectiveness of the 4CMenB vaccine in the Italian pediatric population, including 6 highly populated regions and using both an observational retrospective cohort screening method and a case-control association study. Regional heterogeneity in the immunization schedules represented an excellent opportunity to compare the outcomes associated with different vaccination programs at the population level.

The main outcome of this study was to evaluate the effectiveness of the 4CMenB vaccine in the prevention of serogroup B IMD in the population of children younger than 6 years in 6 Italian regions. Secondary outcomes were the comparison of effectiveness results obtained using 2 different computational methods, the description of serogroup B IMD incidence rates, reduction in IRRs and relative case reduction before and after introduction of the 4CMenB vaccine.

## Methods

This cohort screening study and matched case-control study was approved by the Regional Pediatric Ethical Committee of Tuscany Region on August 31, 2021. All data included in this study were obtained as part of routine clinical activity and evaluated retrospectively and anonymously; therefore, the requirement for informed consent or assent was waived for this study. This study is reported following the Strengthening the Reporting of Observational Studies in Epidemiology (STROBE) reporting guideline for observational studies.

Meningococcal disease is a classified as a notifiable disease in Italy. Serogroup B IMD data from the different regions were obtained from the regional referral centers for the diagnosis of serogroup B IMD and include all notified cases in that specific timeframe in that specific region. Vaccination coverage data were obtained from the regional vaccine-uptake registries. The outcomes associated with 4CMenB introduction in the routine vaccination schedule of 6 Italian regions were explored in the pediatric population conducting both an observational retrospective cohort screening study and a matched case-control study.

### Observational Cohort Screening Study

The cohort screening method allowed a retrospective evaluation of 4CMenB VE and reduction in IRRs through the assessment of incidence and relative case reduction (RCR) of serogroup B IMD before and after introduction of the vaccine in each region. The cohort study was conducted in 3 Italian regions (Piedmont, Tuscany, and Veneto) with similar incidences of serogroup B IMD in the prevaccine era and similar epidemiological surveillance systems but with different vaccination protocols, implemented in different years. The other 3 regions (Apulia, Liguria, and Sicily) were not included in this analysis, since the regional surveillance and reporting systems were subject to significant variations in the study period compared with the other regions and incidence data could not be compared. The different vaccination schedules and the year of implementation of a centralized molecular surveillance system as that of the introduction of the immunization program in each region are summarized in Table 1. The study period spans from the year of implementation of the molecular surveillance program in each region (the first being January 1, 2006) to January 1, 2020. Data obtained after January 1, 2020, were not included in the study to avoid an overestimation bias for VE and reduction in IRRs due to the confounding effects of the population lockdown, social distancing, and widespread use of personal protective equipment during the COVID-19 pandemic. The study was conducted in adherence to the region-specific features customizing the protocol, as detailed in the eMethods in Supplement 1.

**Table 1.** Characteristics of Regional Centralized Molecular Surveillance Program and Vaccination Programs for Invasive Meningococcal Disease and Study Population

Region	Year of implementation		Age of study population, y	Schedule, doses by age, mo	
	Molecular Surveillance Program	4CMenB immunization		At implementation	Actual schedule
Tuscany^a^	2006	2014	0-6	2, 3, 5, and 12	2, 3, 5, and 12
Veneto^a^	2007	2015	0-5	6, 8, and 14	6, 8, and 14
Piedmont^a^	2007	2017	0-3	2, 4, 6, and 14 or 17	3, 5, and 14 or 17 (since September 2018); 2, 4, 12, or 14 (since July 2020)
Liguria	2007	2015	0-5	2, 3, 5, and 14	3, 5, and 14 (since 2019)
Sicily	2011	2015	0-5	2, 3, 5, and 15	2, 3, 5, and 15
Apulia	2007	2014	0-6	2, 3, 5, and 14	2, 3, 5, and 14; 2, 4, and 14 (since 2021)

Abbreviation: 4CMenB, 4-component recombinant protein–based subgroup B meningococcal disease.

^a^ Regions included in the retrospective cohort study.

### Type B IMD Incidence

The IMD incidence rates (IRs; calculated as number of cases per 100 000 children) were calculated as crude IRs in the prevaccine era (reference population) and in the postvaccine era (study population). The denominators used for IR calculations were the numbers of age- and year-specific resident children in each region, as obtained from publicly available data of the Italian National Institute of Statistics. Incidence rates in the postvaccine era were not reported as crude incidence rates but only as age-standardized IRs (ASIRs) weighted on the distribution of IR of the reference population. ASIRs were calculated through direct age standardization, considering the region-specific population of children born before introduction of the 4CMenB vaccine as the reference population and the population of children born after introduction of the 4CMenB vaccine in the same region as the study population. The ASIR was necessary to eliminate the difference in age distribution among reference and study populations.

The incidence rate ratio (IRR) was calculated as incidence rate in postvaccine era divided by incidence rate in prevaccine era. For incidence rate in postvaccine era, the ASIR value was used.

#### VE

Using the formula from Parikh et al^11^ the VE was calculated using the Farrington screening method^20^ with the following formula: 1 − ([PCV / {1 − PCV}]/[MVC / {1 − MVC}]) in which *PCV* is the proportion of children with 4CMenB vaccine among IMD cases and MVC is the mean vaccine coverage in children with the same age in each region. The vaccination coverage data were obtained from the regional vaccination registries.

#### Reduction in IRRs Associated With Vaccination

The impact of 4CMenB vaccination was evaluated as the reduction of IRRs between the prevaccination and the postvaccination eras, calculated as 1 − IRR. This was calculated both as overall reduction in IRRs (the outcomes associated with the 4CMenB vaccine among the entire population, regardless of vaccination status) and reduction in IRRs in the vaccinated population.^10^

The RCR was evaluated as the reduction of the risk of serogroup B IMD expressed by the number of cases prevented in the postvaccination era. The effectiveness, the reduction in IRRs, and the RCR were analyzed separately for each region and then pooled together. Further details are reported in the eMethods in Supplement 1.

### Matched Case-Control Study

The matched case-control study aimed to evaluate the association between 4CMenB vaccination and the event of serogroup B IMD in the postvaccination population (defined exactly as the postvaccination population in the retrospective cohort study) of 6 Italian regions (Apulia, Liguria, Piedmont, Sicily, Veneto, and Tuscany). For each laboratory-confirmed case of serogroup B IMD (excluding those in individuals with medical conditions that put them at increased risk of IMD), 2 controls were matched according to specific criteria, detailed in the eMethods in Supplement 1, together with immunization status definitions. Data from cases and controls were extracted separately from the 6 regions and then analyzed in an aggregate form.

**Figure 1.**
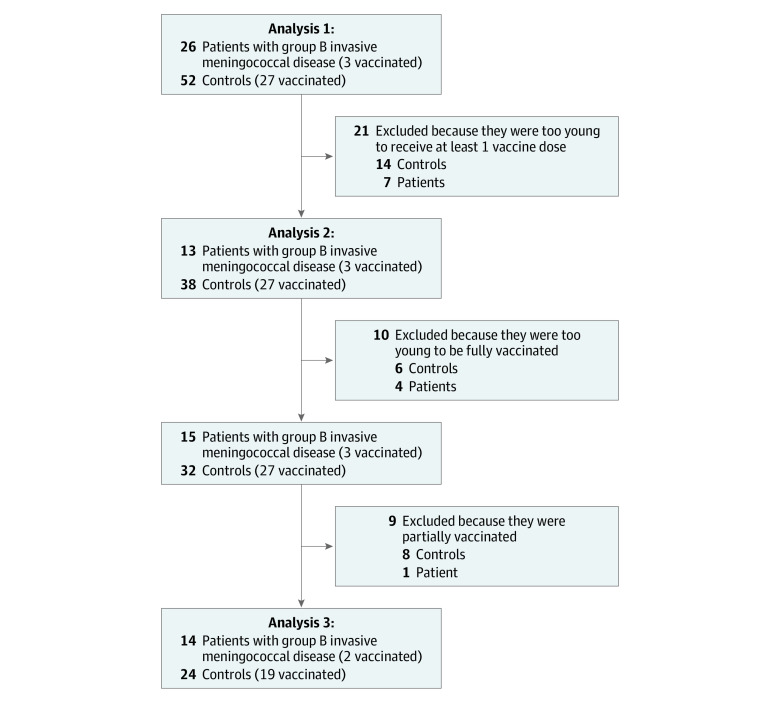
Flowchart of Eligible Individuals in the Matched Case-Control Study by Vaccination Status Analysis 1 includes all children regardless of their age; analysis 2, only children who were old enough to receive at least the first dose of the vaccine; and analysis 3, only children old enough to be fully vaccinated after exclusion of partially immunized children.

Three association analyses were performed, applying different age-inclusion criteria and different definitions for the immunization status. First, all cases and controls were included regardless of the age and vaccination status. In this analysis 1, we evaluated the association between the event serogroup B IMD and the exposure to at least 1 dose of the 4CMenB vaccine (≥14 days previously) (Figure 1). The second analysis included only children who were old enough to receive at least the first dose of the 4CMenB vaccine, and the association analysis was maintained unchanged (event: serogroup B IMD; exposure: having received ≥1 dose of vaccine ≥14 days previously) (Figure 1). For analysis 3, all children who were too young to be fully vaccinated and all those who were partially vaccinated were excluded so that the comparison was carried out between fully vaccinated children and unvaccinated children old enough to be fully immunized (event: serogroup B IMD; exposure: full immunization with vaccine ≥14 days previously) (Figure 1).

Additionally, we evaluated the effectiveness of the 4CMenB vaccine in partially immunized children and in those who had received only 1 dose of 4CMenB.

For each analysis, a difference in the vaccination rates of cases and controls was provided. The calculation of odds ratios (ORs) provided estimates of the VE (calculated as 1 − OR). The 3 association analyses were applied to all 6 regions first and later separately for the 3 regions included in the retrospective cohort screening study (Piedmont, Tuscany, and Veneto) to allow a more accurate comparison of the VE obtained with the 2 methods. Laboratory methods are available in the eMethods in Supplement 1.

### Statistical Analysis

A sample size calculation was performed prior to study initiation to ensure feasibility.^21^ Assuming 50% vaccine uptake in controls, we determined that a minimum of 12 cases with 2 matched controls per case would be required to have a power of 80% (using a 2-sided α = .05) to demonstrate an OR of 0.1 for vaccination.^10^ Data were processed using GraphPad Prism software version 9.5.1 (Dotmatics). We used χ^2^ or Fisher exact test for categorical variables, depending on the number of observations. Two-sided *P* < .05 was considered statistically significant. We used the hybrid Wilson-Brown method to calculate 95% CIs for the incidence rates of the observational cohort screening study and the Baptista-Pike method to calculate 95% CIs for the ORs of the case-control study. Data were analyzed from September 2021 to January 2022.

## Results

### Observational Cohort Screening Study

#### Demographic Data

The resident population on January 1, 2020, in the 3 regions included in the retrospective cohort study was 12 882 905 individuals (21.6% of the total Italian population), distributed as follows: 3 692 555 inhabitants (6.2%) in Tuscany, 4 879 133 inhabitants (8.2%) in Veneto, and 4 311 217 inhabitants (7.2%) in Piedmont. As of January 1, 2020, there were 587 561 children younger than 6 years (20.6% of the total of children in Italy aged <6 years) in these 3 regions. A total of 103 cases of serogroup B IMD (58 [56.3%] males; median [IQR] age, 12.6 [4.9 to 32.3] months) were recorded in the 3 regions during the study period.

#### Incidence

The crude IR of serogroup B IMD in the prevaccine era in Tuscany was 1.54 (95% CI, 1.09-2.18) cases per 100 000 children, while the ASIR in the postvaccination era was 0.40 (95% CI, 0.16-1.03) cases per 100 000 children (IRR, 0.26 [95% CI, 0.09 to 0.74]; *P* = .006). Evaluating cases among vaccinated children only, the ASIR in the postvaccine era was 0.08 (95% CI, <0.01 to 0.44) cases per 100 000 children, a greater difference compared with the prevaccine era (IR, 0.05 [95% CI, 0.01 to 0.37] cases per 100 000 children; *P* < .001).

In Veneto, crude incidence of serogroup B IMD in the prevaccine era was 1.65 (95% CI, 1.20 to 2.27) cases per 100 000 children; in the postvaccine era, the ASIR decreased to 0.91 (95% CI, 0.46 to 1.80) cases per 100 000 children (IR, 0.55 [95% CI, 0.26 to 1.18] cases per 100 000 children; *P* = .12) and to 0.29 (95% CI, 0.05 to 1.05) cases per 100 000 children when considering only cases in vaccinated children, a greater difference compared with the prevaccine era (IR, 0.17 [95% CI, 0.04 to 0.72] cases per 100 000 children; *P* = .004).

In Piedmont, the crude IR in the prevaccine era was 1.55 (95% CI, 0.99 to 2.42) cases per 100 000 children, while the ASIR in the postvaccine era was 1.00 (95% CI, 0.27 to 2.94) cases per 100 000 children (IR, 0.64 [95% CI, 0.19-2.18] cases per 100 000 children; *P* = .60). In Piedmont, no cases of serogroup B IMD were recorded among vaccinated children.

**Figure 2.**
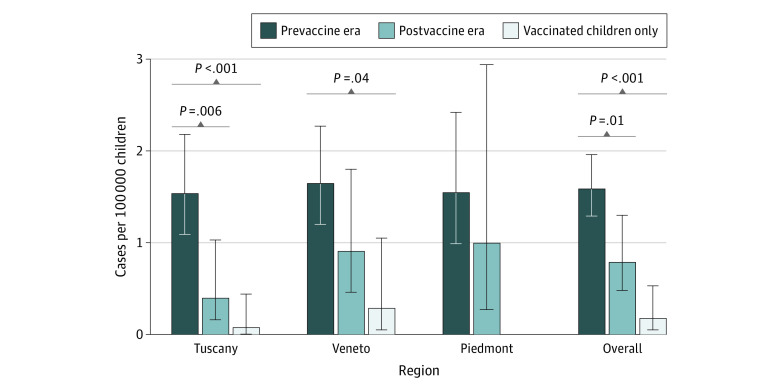
Crude Incidences in the Prevaccine Era and Age-Standardized Incidence Rates in the Postvaccine Era Error bars indicate 95% CIs.

The crude IR in the prevaccine era in all the 3 regions was 1.59 (95% CI, 1.29 to 1.96) cases per 100 000 children, while the ASIR in the postvaccine era was 0.79 (95% CI, 0.48 to 1.30) cases per 100 000 children (IR, 0.50 [95% CI, 0.29 to 0.86] cases per 100 000 children; *P* = .01). Considering only serogroup B IMD cases among vaccinated children in all the 3 regions, the ASIR in the postvaccine era was 0.18 (95% CI, 0.05-0.53) cases per 100 000 children (IR, 0.11 [95% CI, 0.04 to 0.36]; *P* < .001). Crude incidences and ASIR in prevaccine and postvaccine eras in the overall population and in vaccinated children only are presented in Figure 2. RCR results are presented in the eAppendix in Supplement 1.

#### VE

Vaccination coverage data were obtained separately from regional vaccination registries. The vaccination coverage of each region was calculated as the mean of the cohort coverages at age 24 months for the cohorts during 2014 to 2018 in Tuscany, 2015 to 2018 in Veneto, and 2017 to 2018 in Piedmont. The mean vaccination coverage was 83.9% in Tuscany, 83.6% in Veneto, and 81.4% in Piedmont.

The estimated VE was 93.6% (95% CI, 55.4% to 99.1%) in Tuscany, 93.5% (95% CI, 71.7% to 98.5%) in Veneto, and 100% (95% CI, 70.7% to 100%) in Piedmont. The VE considering all 3 regions was 94.9% (95% CI, 83.1% to 98.4%).

#### Reduction in IRRs

The overall reduction in IRR, including both vaccinated and unvaccinated cases, was 0.74 (95% CI, 0.26 to 0.91) in Tuscany, 0.45 (95% CI, −0.18 to 0.74) in Veneto, and 0.36 (95% CI, −1.18 to 0.81) in Piedmont. The reduction in IRR in the vaccinated population was 0.95 (95% CI, 0.63 to 0.99) in Tuscany, and 0.83 (95% CI, 0.28 to 0.96) in Veneto. The overall reduction in IRR in all 3 regions, including both infections among vaccinated and unvaccinated children, was 0.50 (95% CI, 0.14 to 0.71); in analyses restricted to vaccinated children, the reduction in IRR was 0.89 (95% CI, 0.64 to 0.96).

### Matched Case-Control Study

For the case-control study, 6 Italian regions with 23 236 326 inhabitants (39% of the total Italian population) as of January 1, 2020, were included. As of January 1, 2020, there were 1 080 620 children younger than 6 years (38% of the overall population of children age <6 years in Italy). In the postvaccine era, a total of 26 cases of serogroup B IMD (15 [57.7%] male; median [IQR] age, 5.8 [3.3-15.2] months) were recorded in the 6 regions among children born after the vaccine introduction. No patients were excluded according to the exclusion criteria. Two matched controls were identified for each case, for a total of 52 controls. The inclusion flowchart is presented in Figure 1; regional differences are presented in Table 1, and demographic data of cases and controls are detailed in eTable 2 in Supplement 1. Clinical presentation outcomes, including sequelae at hospital discharge; demographics; and laboratory data in vaccinated and unvaccinated cases are presented in Table 2. Of note, the median age of unvaccinated cases was younger than that of vaccinated children (Table 2). Among 23 unvaccinated children with serogroup B IMD, 7 (30.4%) were too young to receive the first dose, and almost half of unvaccinated children (11 children [47.4%]) were too young to be to be fully immunized according to the specific regional vaccination schedule.

**Table 2.** Characteristics of Vaccinated and Unvaccinated Cases

Characteristic	Children with IMD, No. (%)	
	Vaccinated cases (n = 3)	Unvaccinated cases (n = 23)
Age, median (IQR), mo	15.3 (11.2-20.1)	4.4 (3.2-12.8)
Sex		
Male	2 (66.7)^a^	13 (56.5)
Female	1 (33.3)^b^	10 (43.5)
Diagnosis		
Available	3	23
Septicemia and meningitis	0	5 (21.7)
Meningitis	2 (66.7) ^b^	15 (65.2)
Septicemia	1 (33.3)^c^	3 (13.0)
Laboratory method		
Culture	2 (66.7)^a^	6 (26.1)
PCR	1 (33.3)^b^	14 (60.9)
Culture + PCR	0	3 (13.0)
Specimen		
CSF	2 (66.7)^b^	8 (34.8)
Blood	1 (33.3)^c^	5 (21.7)
CSF + blood	0	10 (43.5)
Duration of admission		
Median (IQR), d	14^b^	12.5 (8.5-20.5)
Missing, No.	2	13
Outcome		
Alive with no sequelae^d^	2 (66.7)^a^	17 (73.9)
Alive with sequelae^d^	1 (33.3)^b^^,^^e^	4 (17.4)
Death	0	2 (8.7)
Vaccine status		
Partially vaccinated	1 (33.3)	0
Completely vaccinated	2 (66.7)	0

Abbreviations: CSF, cerebrospinal fluid; PCR, polymerase chain reaction.

^a^ One fully vaccinated child and 1 partially vaccinated child.

^b^ All children in this group were fully vaccinated.

^c^ All children in this group were partially vaccinated.

^d^ Sequelae were evaluated at hospital discharge.

^e^ The patient developed hearing loss but a family history of congenital deafness was reported.

**Figure 3.**
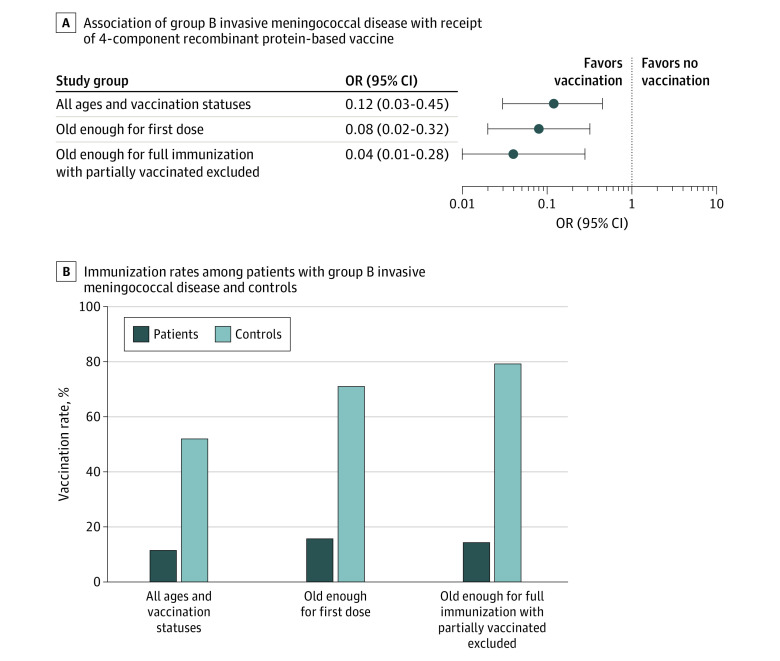
Association of Type B Invasive Meningococcal Disease With Vaccination in the Matched Case-Control Study OR indicates odds ratio.

Of 26 cases of serogroup B IMD, 3 children (11.5%) had received at least 1 vaccine dose; of 52 controls, 27 (51.9%) had received at least 1 vaccine dose (OR, 0.12 [95% CI, 0.03 to 0.45]; *P* < .001; difference, −40.4 [95% CI, −24.1 to −64.7] percentage points). In analyses restricted to children who were old enough to receive at least the first dose of the 4CMenB vaccine, 3 of 19 cases (15.7%) and 27 of 38 controls (71.0%) had received at least 1 vaccine dose (OR, 0.08 [95% CI, 0.02 to 0.32]; *P* < .001; difference, −55.3 [95% CI, −37.9 to −85.3] percentage points). Subsequently, all children who were partially immunized (for whom the extent of protection could not be defined precisely) were excluded from the analysis, and only fully vaccinated children were compared with unvaccinated children old enough to be fully immunized. Two of 14 cases (14.3%) and 19 of 24 controls (79.1%) were fully immunized (OR, 0.04 [95% CI, 0.01 to 0.28]; *P* < .001; difference, −64.8 [95% CI, −47.4 to −101.7] percentage points). The 3 separate analysis, presented in Figure 3A and B, showed a post hoc statistical power greater than 95%. Therefore, the estimate of VE obtained through the case-control study was 87.9% (95% CI, 53.7% to 96.4%) for the whole group and 92.4% (95% CI, 67.6% to 97.9%) when considering only children old enough to receive at least the first 4CMenB dose and considering all children who had received at least 1 dose of 4CMenB as vaccinated. The estimate of VE for fully immunized children was 95.6% (95% CI, 71.7%-99.1%). Of note, 1 of 3 cases (33.3%) were among partially immunized children and 8 of 27 controls (29.6%) were partially immunized; the only case with partial immunization developed IMD at 480 days from the last dose, while for partially immunized controls, the median (IQR) time from last dose at the time of analysis was 33.2 (28.5-248.0) days.

Additional analyses showed that the estimate of VE for children with partial immunization was 91.4% (95% CI, 36.0%-99.3%; *P* = .02; OR, 0.09 [95% CI, 0.01 to 0.64]) and for children who had received only 1 dose of 4CMenB was 100% (95% CI, 60.6%-100%; *P* = .008; OR, <0.01 [95% CI, <0.01 to 0.39]), since no cases of serogroup B IMD were found in children vaccinated with only 1 vaccine dose.

For comparison of the VE obtained with the screening method applied to the retrospective cohort study and the case-control study, a separate association analysis was carried out considering only the 3 regions included in the retrospective cohort study (Piedmont, Tuscany, and Veneto). The estimate of VE was 75.0% (95% CI, 0.2% to 93.3%) for the whole group, 85.7% (95% CI, 25.9% to 96.7%) among children who had received at least 1 dose of 4CMenB, and 91.7% (95% CI, 24.4% to 98.6%) among fully immunized children. Detailed results of case-control study for these 3 regions are presented in the eAppendix in Supplement 1.

## Discussion

This case-control study represents the most comprehensive multiregional evaluation of the effectiveness of 4CMenB vaccination in the pediatric population of Italy. Effectiveness data obtained from a large group of serogroup B IMD cases with the simultaneous application of 2 independent computational methods (screening and case-control) are unique to the literature, and VE were firmly greater than 90% in children old enough to receive the first vaccine dose. Regional differences in the vaccination schedule allowed population-based comparison of outcomes and confirmed the greater efficacy associated with early-start strategies. At the same time, a lack of protection in the very early months of life was apparent even when starting immunization at age 2 months and prompts the identification of extended prevention strategies.

The crude IR of serogroup B IMD in the pre–4CMenB vaccine era was similar in the heterogeneous population of children aged younger than 6 years in the 3 Italian regions operating a similar epidemiological surveillance protocol (Piedmont, Tuscany, and Veneto), at approximately 1.60 cases per 100 000 children. The ASIR calculated in the post–4CMenB vaccine era decreased to 0.79 cases per 100 000 children, with a more significant reduction in Tuscany than in the other 2 regions. The reason for such a difference should be sought in the greater reduction in IRRs associated with vaccination recorded in Tuscany (>70%) than in Veneto (45%) and Piedmont (36%). Indeed, as previously reported,^10^ the reduction in IRRs associated with vaccination was greater where the immunization program started early (as in Tuscany, where vaccination starts at age 2 months). In Veneto, where the first vaccine dose is scheduled after age 6 months, half of serogroup B IMD cases occurred in unvaccinated children and potentially could have been prevented by a vaccination program that started earlier. In Piedmont, the vaccination schedule started at age 3 months and was later adjusted to age 2 months, but as 4CMenB vaccination was implemented in 2017, which was later than the other 2 regions, the postvaccination period was shorter and the population smaller, necessarily resulting in a lower reduction in IRRs than in Tuscany. The overall reduction in IRRs observed in all the 3 regions, including both vaccinated and unvaccinated cases, was 50% and significantly higher when considering vaccinated children only (approximately 90%). These data are not comparable with others reported in literature due to the extreme heterogeneity of study populations, methods and vaccination programs in use (eTable 3 in Supplement 1).^10,11,12,15,16,17,18^

Currently, population-based evidence about VE and reduction in IRRs associated with 4CMenB has been reported in studies conducted in different countries (UK, Australia, Canada, Portugal, and Italy) with heterogeneous methods (screening method, case-control method, cluster-randomized study, Poisson regression model) and across different health care settings and age-groups (eTable 3 in Supplement 1).^10,11,12,15,16,17,18^ The estimates of VE for 4CMenB ranged from 59% to 100% in fully vaccinated cohorts,^10,11,12,13,14,15,16,17,18,19^ being more than 79% in all studies except in 1 conducted in the UK with the screening method^12^ (eTable 3 in Supplement 1), possibly due to a lack of statistical power dependent on the low number of cases in the unvaccinated cohort.^22^ Excluding studies on adolescents and adults, VE for 4CMenB was of 82.9% and 59.1% in UK at 2 different time points,^11,12^ 94.2% in Australia,^16^ and 79% in Canada.^18^ The high variability in the effectiveness data could likely to be due to the variable match of the subcapsular antigens in the type B meningococcal disease strain with the antigens contained in the vaccine,^23^ as suggested by Wang et al,^16^ but it could also be biased by the different computational methods used in each study. Indeed, the screening method is influenced by the consistency of the local surveillance system and by the reliability of the vaccination coverage data. On the other side, the case-control study may be influenced by a selection bias because of the limited number of recruited controls. Therefore, this study used both methods to obtain a more reliable estimate of VE for 4CMenB. The case-control strategy, applied to 6 highly populated Italian regions, found a VE of 92% in children old enough for the first vaccine dose and a VE of 96% in fully immunized children. In the 3 Italian regions with similar epidemiological surveillance systems (Piedmont, Tuscany, and Veneto) the VE in fully-immunized children was 94.9% using the screening method and 91.7% using the case-control method. The similarity of these results, obtained independently with 2 different methods, suggests their reliability and allows comparison with effectiveness data obtained in South Australia (94.2%)^16^ and Canada (100%).^17^

The estimate of VE obtained through the case-control study was greater than 90% also considering only partially immunized children (92%). These data are very likely subject to an overestimation bias due to the limited number of cases but provides clues regarding a potentially protective effect of partial vaccination. In particular, even though the low number of cases prevented us from drawing any definite conclusion, we observed some protection against subgroup B IMD in partially immunized children. Indeed, the only case with partial immunization developed the IMD 480 days from their last dose, while for partially immunized controls, the median time from last dose at the time of analysis was much lower (33 days).

Almost one-third of cases among unvaccinated children were among children too young to receive the first 4CMenB dose and almost a half of them were too young to be vaccinated with at least 2 doses of the vaccine. Even in regions where the first dose of the 4CMenB vaccine is administered at the second month of life, almost 20% of unvaccinated cases were among children too young to receive the first dose of 4CMenB and almost half of them were among children too young to be vaccinated with at least 2 doses. Ladhani et al,^24^ discussing similar findings obtained in another epidemiological setting, proposed to narrow the interval between the first and second dose to 4 weeks to provide earlier protection in the period of life at highest risk for infants. Interestingly, recent data from England shows that the peak age of patients with serogroup B IMD shifted from 5 to 6 months to 1 to 3 months, with approximately half of unvaccinated cases occurring in children too young to receive the first vaccine dose.^25^ In light of this, another possible approach could be to start the vaccination even earlier than age 2 months. Both strategies should be explored by further studies to establish whether a shorter interval between doses, an earlier infant priming, or a combination of both might be most effective in providing protection against serogroup B IMD in the youngest children. Effectiveness data obtained with other vaccines administered in the neonatal period prove that even the newborn can be a potential target of successful immunization.^26^ However, immunogenicity data in younger infants are currently lacking for 4CMenB and should be explored together with the potential role of passive immunization through maternal vaccination, taking into account the sustainability of the different delivery models in specific health care settings.

### Limitations

This study is subject to some limitations. Some, like the limited number of cases, are embedded in the very nature of IMD, which has a low incidence outside of outbreaks. Nevertheless, this study has the largest number of cases on which 2 different methods for the computation of effectiveness were applied, to our knowledge. Other limitations are linked to the retrospective nature of the study and to the heterogeneity of the Italian health care system in terms of regional surveillance and reporting protocols but could be overcome by the use of the case-control method with recruitment of region specific controls.

## Conclusions

This case-control study, the largest in the Italian population, provides further population-based data from before the COVID-19 pandemic confirming the high effectiveness of 4CMenB vaccination in preventing serogroup B IMD in children. Continuous serogroup B IMD surveillance is needed to confirm the high VE reported for 4CMenB so far, in particular after the reduction of COVID-19 prevention strategies. The presented data highlight once more the greater reduction in IRRs associated with early-start vaccination schedules and prompt the identification of further strategies to protect infants in the very first months of life. Also, the study found that the screening and the case-control methods provided comparable estimates of VE. Both methods have specific limitations, and differential use should be tailored to different study settings based on the reliability of the surveillance system and vaccination coverage data, but concomitant use should be pursued when feasible.

## Notes

Supplement 1.
eMethods.
**eAppendix.** Supplementary Results **eTable 1.** Distribution of Neisseria meningitidis Type B (MenB) Invasive Meningococcal Disease (IMD) Cases Based on Age Groups and Year of Diagnosis in Tuscany (2006-2019), Veneto (2007-2019) and Piedmont (2007-2019) **eTable 2.** Epidemiological Data, Final Diagnosis, Microbiological Data, Duration of Hospitalization, Outcome and Vaccination Status of Cases and Controls **eTable 3.** Literature Data on 4CMenB Vaccine Effectiveness and Impact Against Invasive Meningococcal Disease Caused by Serogroup B
eReferences.

Supplement 2.
Members of the Multiregional MenB Study Group

Supplement 3.
Data Sharing Statement
