# Supplementary material for: Four-Component Recombinant Protein–Based Vaccine Effectiveness Against Serogroup B Meningococcal Disease in Italy
Source: JAMA Netw Open. 2023 Aug 18;6(8):e2329678. doi: 10.1001/jamanetworkopen.2023.29678 (PMC10439479; doi:10.1001/jamanetworkopen.2023.29678)
Supplement: Supplement 1. — eMethods. eAppendix. Supplementary Results eTable 1. Distribution of Neisseria meningitidis Type B (MenB) Invasive Meningococcal Disease (IMD) Cases Based on Age Groups and Year of Diagnosis in Tuscany (2006-2019), Veneto (2007-2019) and Piedmont (2007-2019) eTable 2. Epidemiological Data, Final Diagnosis, Microbiological Data, Duration of Hospitalization, Outcome and Vaccination Status of Cases and Controls eTable 3. Literature Data on 4CMenB Vaccine Effectiveness and Impact Against Invasive Meningococcal Disease Caused by Serogroup B eReferences. [file jamanetwopen-e2329678-s001.pdf]

Supplemental Online Content

Lodi L, Barbati F, Amicizia D, et al; Multiregional MenB study group. Four-component recombinant protein–based vaccine effectiveness against serogroup B meningococcal disease in Italy. *JAMA Netw Open*. 2023;6(8):e2329678. doi:10.1001/jamanetworkopen.2023.29678

eMethods.
eAppendix. Supplementary Results
eTable 1. Distribution of Neisseria meningitidis Type B (MenB) Invasive Meningococcal Disease (IMD) Cases Based on Age Groups and Year of Diagnosis in Tuscany (2006-2019), Veneto (2007-2019) and Piedmont (2007-2019)
eTable 2. Epidemiological Data, Final Diagnosis, Microbiological Data, Duration of Hospitalization, Outcome and Vaccination Status of Cases and Controls
eTable 3. Literature Data on 4CMenB Vaccine Effectiveness and Impact Against Invasive Meningococcal Disease Caused by Serogroup B
eReferences.

This supplemental material has been provided by the authors to give readers additional information about their work.

© 2023 Lodi L et al. *JAMA Network Open.*

eMethods

<u>Observational cohort screening study</u>

Regional protocol customization

The oldest age-cohort included is represented on January 1st 2020 by the oldest children that had been vaccine-eligible at birth. Therefore, the study period and the maximum age of inclusion slightly differed across the three regions based on the year of vaccine introduction and molecular surveillance implementation (supplementary table 1). Within the above mentioned region-specific time and age frame, all cases with a laboratory-confirmed diagnosis of MenB IMD were enrolled excluding those occurred in patients with medical conditions at increased risk of IMD, such as primary and secondary immunodeficiencies, immunosuppressive treatments or an history of recurrent invasive bacterial disease. The pre-vaccine population included those patients born before 4CMenB vaccine implementation date, while the post-vaccine population was defined as that population born after the 4CMenB vaccine implementation in each region, through January 1st 2020 (supplementary table 1).

Relative Case Reduction

The Relative Case Reduction was calculated with the following formula:

Standardized Incidence Ratio (SIR) = Observed Cases/Expected Cases

Relative Case Reduction (RCR) = 1-SIR

Expected cases were estimated on the basis of the pre-vaccination incidence rate for each age class.

<u>Matched case-control study</u>

Control inclusion criteria

Controls were subjects of similar age and equal gender of the cases, referred to an Emergency Department or hospitalized in the same region (possibly in the same hospital), for conditions other than meningococcal disease, unspecified meningitis or septicemia, in the 14 days before or after the diagnosis of the paired MenB IMD case. The tolerance of the age difference between a case and the matched control varied depending on the age of the case (for cases aged <2 years,

© 2023 Lodi L et al. *JAMA Network Open.*

the birth date of the control had to be within 14 days of that of the case, for cases aged 2-5 years, within 60 days and for cases aged > 5 years, within 90 days). Epidemiological and microbiological data, final diagnosis, duration of admission, outcome and vaccination status were extracted and recorded in a standardized report form both for the cases and the controls. Clinical records were searched for conditions that would contraindicate the vaccination in each control subject. Only subjects without contraindications were used as controls.

Immunization status definition

Immunization status was defined as:

- FULLY VACCINATED (in order to consider the subject fully vaccinated the time between doses had to be compliant with that defined in the vaccine data sheet as per manufacturer instructions):
  - children <16 months of age who received 2 doses of 4CMenB
  - children ≥ 16 months of age who received at least 2 doses of the vaccine in the first year of life and 1 booster dose after the first year of life
  - children who received two doses of the vaccine after 24 months of age
- PARTIALLY VACCINATED:
  - children who received only 1 dose of 4CMenB vaccine and children who have received more than 1 dose of vaccine but do not meet the "fully vaccinated" criteria. Children who had received only one dose of 4CMenB were also considered as a separate subgroup and analyzed separately.
- NOT VACCINATED:
  - children who did not received any dose of 4CMenB.

Laboratory Methods

Serogroup B meningococcus was detected in blood, cerebrospinal fluid or both using culture, Real Time Polymerase Chain Reaction (RT-PCR) or both. Culture and RT-PCR methodological procedures were applied in compliance with previously described methods.[1,2]

© 2023 Lodi L et al. *JAMA Network Open.*

eAppendix. Supplementary Results

Relative case reduction

In Tuscany the relative case reduction (RCR) was 72% (95% CI 46-89%) and considering the cases occurred in vaccinated subjects only, the RCR reached 93% (95% CI 69-99%).

In Veneto the RCR was 37% (95% CI 17-64%) reaching 84% (95% CI 70-96%) when considering only the cases occurred in vaccinated children.

In Piedmont the RCR was 22% (1-69%) and when including only vaccinated cases reached 100% (95% CI 51-100%) as no case was recorded among vaccinated subjects.

The RCR of the three regions together was 48% (95% CI 31-64%) and 90% (95% CI 75-97%) respectively in all subject and in vaccinated only.

Matched case-control study

Detailed data from the case-control study limited to the three regions (Piedmont, Tuscany and Veneto) included also in the retrospective cohort study are presented below. Three of the 15 cases (20%) and 15 of the 30 controls (50%) had received at least one vaccine dose (OR: 0.250 [95% CI 0.07-0.99]; p=.06; difference: -30% [95% CI -6.5 to -64.0%]).Among unvaccinated children 5 of 12 cases (41.7%) were too young to receive the first vaccine dose. Therefore, when considering only children that were old enough to receive at least the first 4CMenB dose, 3/10 cases (30%) and 15/20 controls (75%) had received at least one vaccine dose (OR: 0.143 [95% CI 0.03-0.74]; p=.04; difference: -45% [95% CI -18.2 to -87.4%]).

All those children that were partially-immunized (for whom the extent of protection could not be defined precisely) were excluded from the analysis and only fully-vaccinated children were compared with unvaccinated children old enough to be fully-immunized. Two of 8 cases (25%) and 12 of 15 controls (80%) were fully-immunized (OR: 0.083 [95% CI 0.01-0.76]; p=.02; difference: -55% [95% CI -29.8 to -103.7%]).

© 2023 Lodi L et al. *JAMA Network Open.*

eTable 1. Distribution of Neisseria meningitidis type B (MenB) invasive meningococcal disease (IMD) cases based on age groups and year of diagnosis in Tuscany (2006–2019), Veneto (2007–2019) and Piedmont (2007-2019).

Red contouring includes year-specific age cohorts included in the study for each region. Yellow boxes refer to subjects born before vaccine introduction (pre-vaccine era or pre-4CMenB), blue boxes refer to subject born after vaccine introduction (post-vaccine era or post-4CMenB). Numbers included in the boxes represent MenB IMD cases occurred in a specific year in a specific age-group.

TUSCANY

	Year of study	2006	2007	2008	2009	2010	2011	2012	2013	2014	2015	2016	2017	2018	2019
Age class															
5-6															
4-5		1				1					1				
3-4					1	1		1	2	1	1				
2-3					1			1						1	
1-2					2	1	1			1			1		
0-1		2	1	3	1	2	1	2	2					2	

VENETO

	Year of study	2006	2007	2008	2009	2010	2011	2012	2013	2014	2015	2016	2017	2018	2019
Age class															
4-5						1	1		1	1			1		
3-4			1				1	1	1	1	1				
2-3			1	1			3	1					1	1	
1-2			2	1		2	1		1				1	1	
0-1				3	2	2	1	3	3		2		1	1	

PIEDMONT

	Year of study	2006	2007	2008	2009	2010	2011	2012	2013	2014	2015	2016	2017	2018	2019
Age class															
2-3						1			1						
1-2					1		1		2	1					
0-1					2	1	2		2	1	1	3	2		1

eTable 2. Epidemiological data, final diagnosis, microbiological data, duration of hospitalization, outcome and vaccination status of cases and controls.

**Characteristic**	**Cases (=26)**	**Controls (=52)**
Age, Median (IQR), months	5.8 (3.3-15.2)	5.9 (3.4-15.5)
Sex, (%)		
Available	26	52
Male	15 (57.7)	30 (57.7)
Female	11 (43.3)	22 (43.3)
Diagnosis, (%)		
Available	26	52
Septicemia and meningitis	5 (19.2)	0
Meningitis	17 (65.4)	0
Septicemia	4 (15.4)	0
Gastroenteritis	0	9 (17.3)
URTI	0	14 (26.9)
Trauma	0	7 (13.5)
Bronchiolitis	0	3 (5.8)
Otitis	0	2 (3.8)
Asthma	0	1 (1.9)
UTI	0	5 (9.6)
Conjunctivitis	0	1 (1.9)
Other	0	10 (19.2)
Laboratory method		
Available	23	n.a.
Culture	6 (26.1)	
PCR	14 (60.9)	
Culture + PCR	3 (13.0)	
Specimen		
Available	26	n.a.
CSF	10 (38.5)	
Blood	6 (23.1)	
CSF + Blood	10 (38.5)	
Duration of admission		
Available	11	52
Median (IQR), days	13 (9-20)	1 (0-4)
Outcome, (%)		
Available	26	n.a.
Alive with no sequelae[a]	19 (73.1)	
Alive with sequelae[a]	5 (19.2)	
Death	2 (7.7)	
Vaccine status		
Available	26	52
Not vaccinated	23 (88.5)	25 (48.1)
Vaccinated	3 (11.5)	27 (51.9)
Partially vaccinated	1 (33.3)	8 (29.6)
Fully vaccinated	2 (66.7)	19 (70.4)

Abbreviations. CSF: cerebrospinal fluid; IQR: interquantile range; n.a.: not applicable; URTI: upper respiratory tract infection; UTI: urinary tract infection; PCR: polymerase chain reaction.
[a] sequelae were evaluated at hospital discharge.

© 2023 Lodi L et al. *JAMA Network Open.*

eTable 3. Literature data on 4CMenB vaccine effectiveness and impact against invasive meningococcal disease caused by serogroup B.

Authors	Country	Region	Study Method	Study Period	Population number	Age group	Effectiveness	Impact
**Parikh et al.** [3]	UK	England	Screening method	2011-15 vs 2015-16	Average 74 vs 37 cases	0-2 years	After a single dose: 22.0% (-105 - 67.1)<br><br>After 2 doses: 82.9% (24.1 - 95.2)	50%
**Ladhani et al.** [4]	UK	England	Screening method	2013-14 vs 2015-18	187 cases (147 old enough to receive at least one dose)	0-4 years	After a single dose: 24.1% (37.6 - 58.2)<br><br>After 2 doses: 52.7% (33.5 - 83.2)<br><br>Fully vaccinated: 59.1% (31.1 - 87.2)	75%
**Argante et al.** [5]	UK	England	Poisson regression model	2011-14 vs 2015-18	n.a.	0 month-older than 44 years	After a single dose: 33.5% (12.4 - 49.7)<br><br>After 2 doses: 78.7% (71.5 - 84.5)<br><br>Fully vaccinated: 80.1% (70.3 - 86.7)	n.a.
**Marshall et al.** [6]	Australia	South Australia	Cluster randomized study	2015-16 vs 2017-19	12 vs 0 cases	15-18 years	100%	n.a.
**McMillan et al.** [7]	Australia	South Australia	Cluster randomized study	2003-16 vs 2017-19	1-10 cases/year vs 5 cases in 2017-18 and 1 case in 2018-19	16-19 years	n.a.	71%
**Wang et al.** [8]	Australia	South Australia	Screening method	2012-18 vs 2018-20	2 cases<br><br>4<br><br>4<br><br>2 cases	12 weeks-11 months<br><br>1 year<br><br>2 years<br><br>15-18 years	94.2% (36.6 - 99.5)	60%<br><br>21%<br><br>25%<br><br>73%
			Case-control method	2018-20	2 cases<br><br>2 cases	0-2<br><br>15-21 years	94.7% (40.3 - 99.5)<br><br>100%	n.a.
**De Wals et al.** [9]	Canada	Quebec, Saguenay-Lac-Saint-Jean region	Poisson regression model	2006-14 vs 2014-16	56 vs 0 cases<br><br>19 vs 2 cases	≤20<br><br>>20 years	100%	100%<br><br>67%
**Deceuninck et al.** [10]	Canada	Quebec, Saguenay-Lac-Saint-Jean region	Poisson regression model	2006-14 vs 2014-18	56 vs 1 case<br><br>19 vs 4 cases	≤20<br><br>>20 years	79% (-231 - 99)	96%<br><br>59%

© 2023 Lodi L et al. *JAMA Network Open.*

**Rodrigues et al.** [11]	Portugal	n.a.	Case-control method	2014-2019	98 cases (82 old enough to receive at least one dose, 69 to be fully vaccinated)	0-5 years	At least 1 dose: 82% (56 - 92)<br><br>Fully vaccinated: 79% (45 - 92)	n.a.
**Azzari et al.** [12]	Italy	Tuscany	Screening method	2006-13 vs 2014-18	31 vs 4 cases	0-5 years	93.6% (55.4 - 99.1)	68%<br><br>94% (only vaccinated children)
		Veneto	Screening method	2007-14 vs 2015-18	34 vs 7 cases	0-4 years	91.0% (59.9 - 97.9)	31%<br><br>90% (only vaccinated children)
**Present Study**	Italy	Tuscany<br>Veneto<br>Piedmont	Screening method	2006-2020	88 vs 15 cases	0-6<br>0-5<br>0-3<br>years	94.9% (83.1 - 98.4)	50%<br><br>89% (only vaccinated children)
		Tuscany<br>Veneto<br>Piedmont<br>Liguria<br>Sicily<br>Apulia	Case-control method	2014-20<br>2015-20<br>2017-20<br>2015-20<br>2015-20<br>2014-20	26 cases (19 old enough to receive at least one dose, 14 to be fully vaccinated)	0-6<br>0-5<br>0-3<br>0-5<br>0-5<br>0-6<br>years	At least 1 dose: 92% (68 - 98)<br><br>Fully vaccinated: 96% (72 - 99)	n.a.

© 2023 Lodi L et al. *JAMA Network Open.*

eReferences

1. Azzari C, Moriondo M, Nieddu F, et al. Underestimation of invasive meningococcal disease in Italy. *Emerg Infect Dis.* 2016;22(3):469–75.
2. Guiducci S, Moriondo M, Nieddu F, et al. Culture and Real-time Polymerase Chain reaction sensitivity in the diagnosis of invasive meningococcal disease: Does culture miss less severe cases? *PLoS ONE.* 2019;14(3):e0212922.
3. Parikh SR, Andrews NJ, Beebeejaun K, et al. Effectiveness and impact of a reduced infant schedule of 4CMenB vaccine against group B meningococcal disease in England: a national observational cohort study. *Lancet.* 2016;388(10061):2775–82.
4. Ladhani SN, Andrews N, Parikh SR, et al. Vaccination of Infants with Meningococcal Group B Vaccine (4CMenB) in England. *N Eng J Med.* 2020;382(4):309–17.
5. Argante L, Abbing-Karahagopian V, Vadivelu K, Rappuoli R, Medini D. et al. A re-assessment of 4CMenB vaccine e effctiveness against serogroup B invasive meningococcal disease in England based on an incidence model. *BMC Infect Dis.* 2021;21(1):1244.
6. Marshall HS, McMillan M, Koehler AP, et al. Meningococcal B Vaccine and Meningococcal Carriage in Adolescents in Australia. *N Eng J Med.* 2020;382(4):318–27.
7. McMillan M, Wang B, Koehler AP, Sullivan TR, Marshall HS. Impact of Meningococcal B Vaccine on Invasive Meningococcal Disease in Adolescents. *Clin Infect Dis.* 2021;73(1):e233–37.
8. Wang B, Giles L, Andraweera P, et al. Effectiveness and impact of the 4CMenB vaccine against invasive serogroup B meningococcal disease and gonorrhoea in an infant, child, and adolescent programme: an observational cohort and case-control study. *Lancet Infect Dis.* 2022;22(7):1011–20.
9. De Wals P, Deceuninck G, Lefebvre B, et al. Impact of an Immunization Campaign to Control an Increased Incidence of Serogroup B Meningococcal Disease in One Region of Quebec, Canada. *Clin Infect Dis.* 2017;64(9):1263–67.
10. Deceuninck G, Lefebvre B, Tsang R, Betala-Belinga JF, De Serres G, De Wals P. Impact of a mass vaccination campaign against Serogroup B meningococcal disease in the Saguenay-Lac-Saint-Jean region of Quebec four years after its launch. *Vaccine.* 2019;37(31):4243–45.
11. Rodrigues FMP, Marlow R, Simões MJ, Danon L, Ladhani S, Finn A. Association of Use of a Meningococcus Group B Vaccine With Group B Invasive Meningococcal Disease Among Children in Portugal. *JAMA.* 2020;324(21):2187–94.
12. Azzari C, Moriondo M, Nieddu F, et al. Effectiveness and Impact of the 4CMenB Vaccine against Group B Meningococcal Disease in Two Italian Regions Using Different Vaccination Schedules: A Five-Year Retrospective Observational Study (2014–2018). *Vaccines.* 2020;8(3):469.

© 2023 Lodi L et al. *JAMA Network Open.*
