# Supplementary material for: Four-Component Recombinant Protein–Based Vaccine Effectiveness Against Serogroup B Meningococcal Disease in Italy
Source: JAMA Netw Open. 2023 Aug 18;6(8):e2329678. doi: 10.1001/jamanetworkopen.2023.29678 (PMC10439479; doi:10.1001/jamanetworkopen.2023.29678)
Supplement: Supplement 2. — Members of the Multiregional MenB Study Group [file jamanetwopen-e2329678-s002.pdf]

Supplemental Online Content: Nonauthor Collaborators
*First name, last name, and suffix (if applicable) are required and will appear in PubMed.

*Group Name(s): Multiregional MenB Study Group							
***First Name and Middle Initial(s)**	***Last Name**	***Suffix (eg, Jr, III)**	Academic Degrees	Institution	Location (city, state/province, country)	Role or Contribution, eg, chair, principal investigator	Group (if more than 1 Group listed in the byline) and/or Subgroup (eg, Steering Committee)
Mario	Palermo		MD	Sicilian Health Department	Palermo, Italy.	Non-author contribution to data collection.	
Girolama	Bosco		MD	Local Health Authority (LHA) Agrigen	Agrigento, Italy.	Non-author contribution to data collection.	
Rosanna	Milisenna		MD	LHA Caltanissetta	Caltanissetta, Italy.	Non-author contribution to data collection.	
Mario	Cuccia		MD	LHA Catania	Catania, Italy.	Non-author contribution to data collection.	
Franco	Belbruno		MD	LHA Enna	Enna, Italy.	Non-author contribution to data collection.	
Giulia Tarabini	Castellani		MD	LHA Messina	Messina, Italy.	Non-author contribution to data collection.	
Claudio	D'Angelo		MD	LHA Palermo	Palermo, Italy.	Non-author contribution to data collection.	
Giuseppe	Ferrera		MD	LHA Ragusa	Ragusa, Italy.	Non-author contribution to data collection.	
Lia	Contrino		MD	LHA Siracusa	Siracusa, Italy.	Non-author contribution to data collection.	
Gaspare	Canzoneri		MD	LHA Trapani	Trapani, Italy.	Non-author contribution to data collection.	
Onofrio	Mongelli		MD	Apulian Regional Health Authority	Bari, Italy.	Non-author contribution to data collection.	
Nehludoff	Albano		MD	Apulian Regional Health Authority	Bari, Italy.	Non-author contribution to data collection.	
Domenico	Lagravinese		MD	LHA Bari,	Bari, Italy.	Non-author contribution to data collection.	
Riccardo	Matera		MD	LHA Barletta-Andria-Trani	Andria, Italy.	Non-author contribution to data collection.	
Stefano	Termite		MD	LHA Brindisi	Brindisi, Italy.	Non-author contribution to data collection.	
Giovanni	Iannucci		MD	LHA Foggia	Foggia, Italy.	Non-author contribution to data collection.	
Alberto	Fedele		MD	LHA Lecce	Lecce, Italy.	Non-author contribution to data collection.	
Michele	Conversano		MD	LHA Taranto	Taranto, Italy.	Non-author contribution to data collection.	
Irene	Amoruso		MD	Department of Cardiac, Thoracic, Vas	Padua, Italy.	Non-author contributic	Multiregional MenB study group
Tatjana	Baldovin		MD	Department of Cardiac, Thoracic, Vas	Padua, Italy.	Non-author contributic	Multiregional MenB study group
Silvia	Cocchio		MD	Department of Cardiac, Thoracic, Vas	Padua, Italy.	Non-author contributic	Multiregional MenB study group
Nicole	Bonaccorso		MD	Department of Health Promotion Sci	Palermo, Italy.	Non-author contributic	Multiregional MenB study group
Martina	Sciortino		MD	Department of Health Promotion Sci	Palermo, Italy.	Non-author contributic	Multiregional MenB study group

Supplemental Online Content: Nonauthor Collaborators

*First name, last name, and suffix (if applicable) are required and will appear in PubMed.

***First Name and Middle Initial(s)**	***Last Name**	***Suffix (eg, Jr, III)**	Academic Degrees	Institution	Location (city, state/province, country)	Role or Contribution, eg, chair, principal investigator	Group (if more than 1 Group listed in the byline) and/or Subgroup (eg, Steering Committee)
Arianna	Conforto		MD	Department of Health Promotion Sci	Palermo, Italy.	Non-author contributic	Multiregional MenB study group
Susanna	Masiero		MD	Department of Woman's and Child's	Padua, Italy.	Non-author contributic	Multiregional MenB study group
Daniela	Lombardi		MD	Regional Epidemiology reference ser	Alessandria, Italy.	Non-author contributic	Multiregional MenB study group
Elisa	Di Maggio		MD	Hygiene Unit, Policlinico Foggia Hosp	Foggia, Italy.	Non-author contributic	Multiregional MenB study group
Martina	Meola		MD	Hygiene Unit, Policlinico Foggia Hosp	Foggia, Italy.	Non-author contributic	Multiregional MenB study group
Silvia	Boscia		MSc	Laboratory of Immunology and Mole	Florence, Italy.	Non-author contributic	Multiregional MenB study group
Michele	Tonon		MD	Regional Directorate of Prevention, F	Venice, Italy.	Non-author contributic	Multiregional MenB study group
Marzio	Masini		MD	Meyer Children's Hospital IRCCS	Florence, Italy.	Non-author contributic	Multiregional MenB study group
Maria Francesca	Piazza		MD	Regional Health Agency of Liguria (A.	Genoa, Italy.	Non-author contributic	Multiregional MenB study group
Camilla	Sticchi		MD	Regional Health Agency of Liguria (A.	Genoa, Italy.	Non-author contributic	Multiregional MenB study group
