# Supplementary material for: Four-Component Recombinant Protein–Based Vaccine Effectiveness Against Serogroup B Meningococcal Disease in Italy
Source: JAMA Netw Open. 2023 Aug 18;6(8):e2329678. doi: 10.1001/jamanetworkopen.2023.29678 (PMC10439479; doi:10.1001/jamanetworkopen.2023.29678)
Supplement: Supplement 3. — Data Sharing Statement [file jamanetwopen-e2329678-s003.pdf]

Data Sharing Statement

Lodi. Four-Component Recombinant Protein–Based Vaccine Effectiveness Against Serogroup B Meningococcal Disease in Italy. *JAMA Netw Open*. Published August 18, 2023. doi:10.1001/jamanetworkopen.2023.29678

Data
Data available: Yes
Data types: Deidentified participant data
How to access data: lorenzo.lodi@unifi.it
When available: With publication

Supporting Documents
Document types: None

Additional Information
Who can access the data: Researchers whose proposed use of the data has been approved.
Types of analyses: For a specified purpose
Mechanisms of data availability: After approval of a proposal
